# Optimal Sense-Making and Resilience in Times of Pandemic: Integrating Rationality and Meaning in Psychotherapy

**DOI:** 10.3389/fpsyg.2021.645926

**Published:** 2021-03-30

**Authors:** Pninit Russo-Netzer, Matti Ameli

**Affiliations:** ^1^Department of Advanced Studies, Achva Academic College, University of Haifa, Haifa, Israel; ^2^Private Practice, Valencia, Spain

**Keywords:** logotherapy, rational emotive behavior therapy, rationality, meaning, resilience, self-transcendence, COVID-19 pandemic

## Abstract

The global COVID-19 pandemic has triggered a wide variety of psychological crises worldwide. In order to respond rapidly and efficiently to the complex challenges, mental health professionals are required to adopt a multidimensional and integrative view. Rational Emotive Behavior Therapy (REBT) founded by Albert Ellis promotes rationality and self-acceptance. Logotherapy, pioneered by Viktor Frankl potentiates meaning and resilience. Both approaches are complementary and mutually enriching. The goal of this paper is to propose an integrative model of “optimal sense-making,” a concept that combines both rationality and meaning, as well as the role of self-transcendence and healthy negative emotions. The model offers a theoretical and clinical foundation for efficient and effective psychological intervention plans for those affected by the pandemic. Along with theoretical background, illustrating case studies are presented to support potential application of the integrative model to affected individuals as well as the work of first-line health professionals during these times of pandemic. Implications are considered for utilizing theoretical and applied insights from the model to cultivate resilience in face of adversity and suffering.

## Introduction

The unprecedented coronavirus pandemic caught us by surprise and has caused so far over 1,250,000 deaths worldwide. Few mental health professionals have experience dealing with the psychological consequences of such devastating pandemic. This new and challenging situation requires flexibility, creativity, and integrative attitude from mental health professionals.

Rational emotive behavior therapy (REBT), the pioneering form of cognitive-behavioral therapy developed by Albert Ellis in the 1950's and Logotherapy, a meaning-based psychotherapy proposed by Viktor Frankl in the 1930's are philosophically based and empirically-supported. Their integration could lead to the marriage of rationality and meaning, enabling a more stable and profound foundation to counteract catastrophism and despair in the face of the tragedy. For example, Dryden (2020) highlighted the importance of rationality to avoid adding horror to the crisis. Complementarily, the importance of meaning-making has been emphasized as a resource in coping with challenging life circumstances such as adversity, crisis, and trauma (e.g., Janoff-Bulman and Yopyk, 2004; Damon, 2008; Melton and Schulenberg, 2008; Linley and Joseph, 2011; Park, 2013; Czekierda et al., 2017). According to the meaning-making model, for example, perceived discrepancies between appraised meaning of a particular situation and global meaning (i.e., general orienting systems of beliefs and goals) create distress, which generates meaning-making efforts to reduce it. Combining the two approaches of rationality and meaning, Ameli (2020) proposed initial ideas on how to use logotherapy as an adjunct to REBT to help therapists deal with potential personal challenges during the pandemic. The present paper takes these directions forward to offer an integrative clinical model of “optimal sense-making,” based on a brief overview of both REBT and logotherapy, and illustrated though clinical examples of case studies related to the current pandemic situation.

### Overview of REBT

The American psychologist Albert Ellis (1913–2007) founded Rational Emotive Behavior Therapy (REBT) in 1955. REBT is considered the first form of Cognitive-Behavioral Therapy (CBT) and Ellis is considered the grandfather of CBT, and a major contributor to the cognitive paradigm shift in the field of clinical psychology (David, 2015). Ellis (1962) was highly influenced by Greek Stoic philosophers such as Epictetus and Marcus Aurelius.

Ellis proposed the ABC (or ABCDE) model to conceptualize the role of thinking process in emotional disturbance. The *A* stands for Activating events or Adversity, the *B* stands for Beliefs that includes functional or rational beliefs (RBs) and dysfunctional or irrational beliefs (IBs), and the *C* stands for emotional, behavioral and cognitive Consequences. Those consequences could also become themselves activating events (A), producing secondary or meta-consequences (e.g., anxiety about being anxious or depression about being depressed). Clients learn through the process of therapy to actively and vigorously dispute (D) their irrational beliefs in order to generate effective new philosophies (E) based on healthy, functional and adaptive beliefs. It is important to note that although REBT emphasizes B as the main mediator between A and C, it views cognition, emotion, behavior as interconnected, and interactive processes, influencing each other (Dryden et al., 2010; Ellis and Ellis, 2011; DiGiuseppe et al., 2014).

Ellis believed that a dogmatic and rigid philosophy of demandingness based on absolutistic beliefs expressed in terms of “musts,” “should,” “oughts,” and “have to's” toward self, others, and life or the world was at the heart of psychological disturbance, leading to three other types of irrational beliefs: awfulizing (an extreme exaggeration of the negative consequences of a situation), frustration intolerance (demanding comfort and ease at all times and not tolerating discomfort), and global evaluation of worth of self or others (the idea that human being are ratable and some are worthless or less valuable than others) (DiGiuseppe et al., 2014). While rigid and dogmatic beliefs are at the core of psychological disturbance, psychological flexibility based on relativism, non-dogmatic preferences and unconditional acceptance is a key feature of psychological health (Dryden et al., 2010; DiGiuseppe et al., 2014). Given that REBT promotes a rational life philosophy emphasizing unconditional self-acceptance, unconditional other acceptance and unconditional life acceptance (Ellis and Ellis, 2011), it may be particularly relevant to the current pandemic situation because it helps counteract the sense of guilt, shame and blame in affected individuals, paving the path toward greater sense of comprehension, purpose, and compassion.

REBT distinguishes between unhealthy/maladaptive and healthy/adaptive negative emotions. Healthy negative emotions such as concern, annoyance, sadness, disappointment, and regret or remorse are based on rational beliefs whereas unhealthy negative emotions such as anxiety, depression, clinical anger, shame, and guilt are based on irrational beliefs. For example, concern is based on the belief “I hope nothing bad happens to me but I am not immune from it and if it happens, it would be unfortunate not terrible.” In contrast, anxiety is associated with the belief “Nothing bad should happen to me and if it does it would be terrible.” REBT only targets unhealthy and maladaptive negative emotions (Dryden et al., 2010; Dryden, 2012; DiGiuseppe et al., 2014). Dryden (2020) views the concept of healthy negative emotions as one of the strength of the REBT model. In face of great loss or tragedy, “taking the horror out of the tragedy” (Dryden, 2020, p. 300) could be liberating for clients, since they can still feel painful and embrace strong healthy negative emotions.

REBT is a multimodal approach used in different cultures, which has integrated a variety of cognitive, emotive and behavioral techniques that can be used with a wide range of clients (Ellis and MacLaren, 2005). The main goal of REBT is to identify, dispute and modify rigid, irrational and unhealthy philosophies based on absolutistic demands, and a lack of unconditional acceptance of self, others and life/world into a flexible and healthy philosophy based on preferences and unconditional acceptance (Ellis and Ellis, 2011). *Disputing*, the best-known method of REBT, aims at helping clients to identify their core irrational beliefs. Functional disputes question the helpfulness of the client's belief and the resulting behaviors and emotions, empirical disputes are centered in finding out the if there is empirical evidence that support the client's belief, and logical disputing is focused on questioning the illogical absolutistic demands instead of preferences. A variety of questioning styles such as Socratic, didactic or humorous are used (Ellis and MacLaren, 2005; DiGiuseppe et al., 2014). REBT is an active-directive therapy which emphasizes unconditional acceptance of clients despite their self-defeating patterns in order to facilitate and encourage unconditional self-acceptance (DiGiuseppe et al., 2014).

REBT is a scientific and evidence-based form of psychotherapy that has been found effective for a large spectrum of clinical psychiatric disorders (depression, anxiety disorders, addiction, eating disorders etc.) and populations (David et al., 2005). Results of recent meta-analysis related to the relationship between both irrational and rational beliefs and psychological distress show a positive correlation between irrational beliefs and various types of psychological distress such as anxiety, depression, general distress, anger, and guilt (Vîsla et al., 2016), and a negative association between rational beliefs and psychological distress, with the strongest association being for unconditional acceptance beliefs (Oltean and David, 2018). A thorough review and meta-analysis of REBT over the last 50 years demonstrates the efficacy and effectiveness of REBT interventions in the format of psychotherapy, psycho-education, or counseling for various conditions regardless of age, clinical status, and delivery format (David et al., 2018). Yet, the current pandemic situation calls for more breadth and depth in responding to the crisis and its implications. The COVID 19 virus has affected humanity in all countries, cultures, and civilizations. Consequently, humanity is facing a sense of despair or meaninglessness, and many question the meaning of their lives. Essentially, the disruption of routine, social distancing, uncertainty, isolation and loneliness have shaken and violated important aspects of people's sense of meaning, such as comprehension, purpose, and mattering (cf. George and Park, 2016; Martela and Steger, 2016) and has influenced their mental and physical well-being (see De Jong et al., 2020). It is thus of particular importance to go beyond rationality and to integrate the framework of meaning to not only discover a “why” to say yes to life in spite of the crisis but to also gain resilience and even grow in the face of adversity. A Logotherapy- enhanced REBT has the potential to offer a deep, hopeful and powerful therapy in the long term. In this context, for example, Frankl (1986) proposed the term “monoanthropism” or shared humanity, which can be valuable for both therapists and clients to familiar with, to understand that they are not alone in this situation.

### Overview of Logotherapy

The Austrian neurologist, psychiatrist, and doctorate in philosophy Viktor Frankl (1905–1997) pioneered Logotherapy during the 1930's. It is generally defined as an empirically based meaning-centered approach to psychotherapy. Frankl (1969) envisioned logotherapy as an undogmatic system of therapy, open to its own evolution as well as collaboration with other psychotherapeutic orientations. It has been called the “third Viennese School of Psychotherapy” (the first one being Freud's Psychoanalysis and the second Adler's individual Psychology).

Logotherapy envisions the human person in three overlapping dimensions: somatic-physical, psychological, and noetic-spiritual. Frankl (1969) referred to the spiritual dimension as “noetic” to avoid religious connotations. The noetic dimension is considered the healthy of authentically human phenomena and includes qualities such as self-distancing, self-transcendence, humor, values, imagination, love, and gratitude. In contrast with the first two dimensions where our reactions are often automatic, in the third dimension, we can choose how to behave (Lukas, 1998). Intentionality is the key factor that makes human beings unpredictable. Frankl's theory is based on the premise that human beings are motivated by a “will to meaning,” an inner pull to discover meaning in life. The fundamental tenets of logotherapy are freedom of will, will to meaning and meaning in life (Frankl, 1969). *Freedom of will* asserts that human beings have the freedom to choose their response within the limits of given possibilities, under all life circumstances. *Will to meaning* points out that the main motivation of human beings is to search for the meaning and purpose in their lives. *Meaning in life* highlights that life has meaning under all circumstances, even in unavoidable suffering and misery.

We can discover meaning in life in three different ways known as the categorical values. These categorical values are comprised of the *creative values*, the *experiential values*, and the *attitudinal values* (Frankl, 1959/1984). The creative values consist of what we give to the world, like accomplishing a task, creating a work, or doing a good deed. The experiential values are what we take from the world, like the experience of truth, beauty and love toward another human being. The attitudinal values reflect the stand we take toward an unchangeable situation or unavoidable suffering (Frankl, 1959/1984). *Tragic optimism* (Frankl, 1959/1984) refers to remaining optimistic through hope, faith and love in spite of the *tragic triad* of pain, guilt, and death. This is based on the principle that life is meaningful under all circumstance and the human capacity to make the best of any given situation by turning creatively the negative aspects into constructive ones (Frankl, 1959/1984; Lukas, 1998).

The three main techniques used in logotherapy are paradoxical intention (using self-distancing through humor to counteract anticipatory anxiety), dereflection (shifting the focus of attention toward meaning through self-transcendence), and attitude modification (challenging a negative attitude by activating the will to meaning through Socratic dialogue).

The goal of the logotherapist is to tap into unique human capacities such as intentionality, responsibility and freedom of choice, and to broaden clients' visual scope to help them discover and actualize the meaning potentials in their lives (Ameli, 2016a,b). Logotherapy focuses both on the client's “current positives” (assets and strengths) and “future potentials” or possibilities for expansion (Lukas, 1998). The logotherapist awakens and mobilizes clients' inner resources and orients them toward areas where meaning can be found in their unique situation (Marshall and Marshall, 2017). The logotherapist is active during the therapy session, pointing out incoherencies, sharing ideas and disagreeing with clients when their values are not adjusted to reality (Lukas, 1998).

In clinical practice, logotherapy has been found useful with problems such as depression, anxiety, alcohol/drug addiction, psychosis, grief, and despair associated with incurable disease (Schulenberg et al., 2008; Marshall and Marshall, 2017).

A large number of research studies have been conducted to validate the main concepts, constructs and tools used in logotherapy (Batthyany and Guttmann, 2006). The concept of meaning has been validated by research in logotherapy. The Purpose in Life test (PIL), a 20-item psychometric tool developed by Crumbaugh and Maholick (1964) is the oldest and most investigated instrument that measures the degree to which a person experiences a sense of personal meaning. The PIL is consistent with the logotherapy postulate with a high degree of reliability, shows positive correlations with items such as self-control, life satisfaction, self-acceptance, emotional stability and resilience, and is correlated negatively with anxiety and depression (Melton and Schulenberg, 2008). The Purpose in Life test-Short Form (PIL-SF; Schulenberg et al., 2011) is a brief four-item valid and reliable version of the PIL presenting unique psychometric contributions beyond other meaning assessment tools. Recently, Shoshani and Russo-Netzer (2017) developed the Meaning in Life in Children Questionnaire (MIL-CQ), a 21-item instrument that measures the presence and sources of meaning in life in children, based on Frankl's categorical values, and Russo-Netzer (2018a) proposed the construct of “prioritizing meaning,” positively associated with well-being.

Meaning-centered individual and group therapy psychotherapy programs have also been found as highly effective and empirically validated in various settings and populations (e.g., Breitbart and Masterson, 2016; Southwick et al., 2016; Weathers et al., 2016). These ideas and tools appear to be especially relevant in the context of the present COVID crisis.

### Integration of REBT With Logotherapy

REBT and Logotherapy share many similarities and present a high degree of compatibility. Ellis (1985) referred to Frankl, highlighting the importance of values in psychotherapy and (Frankl, 2004, p. 83) insisted on the importance of questioning the rational validity of clients' philosophy of life.

Hutchinson and Chapman (2005) and Lewis (2009) have proposed innovative ideas to integrate REBT with logotherapy at the clinical level. Hutchinson and Chapman (2005) argue that cognitive shifts in REBT and logotherapy are complementary and propose to augment REBT disputation techniques with logotherapeutic concepts such as hope, faith and optimism, in addition to reason. Since REBT differentiates between healthy and unhealthy negative emotions, Hutchinson and Chapman (2005) believe that introducing the concept of meaning could help clients to better tolerate the adaptive negative emotions such as sadness, frustration, or disappointment by addressing their potential inherent meaning. At the metacognitive level, logotherapy-enhanced REBT could be more efficient to decrease and counteract secondary disturbance in the form of rumination, constant self-evaluation or excessive reflection on the rationality of one's thinking, using the technique of dereflection.

Considering the similarities and contrasts between REBT and logotherapy, both approaches aim at replacing unhelpful and detrimental beliefs with beneficial ones (Lewis, 2009). In REBT detrimental beliefs are irrational beliefs and reason is used to replace them with rational ones while in logotherapy, the process of meaning discovery is employed to help clients choose beneficial attitudes, defined as consistent beliefs. Lewis (2009) points out that in the language of REBT, beneficial attitudes could correspond to “meaningful attitudes” while detrimental beliefs could be labeled as “meaningless” or “nihilistic beliefs.” Thus, beliefs that are both rational and meaningful have the advantage to lead to self-transcendence and may produce greater benefits for clients.

## Proposal for An Integrative Model: Optimal Sense-Making

Building on the ideas presented by Hutchinson and Chapman (2005) and Lewis (2009), we propose to include an explicit and systematic exploration of meaning in the framework of REBT, in order to promote resilience in the face of the current pandemic.

At the theoretical level, REBT offers a rational and empirically supported explanation of psychopathology by emphasizing the four key irrational beliefs (i.e., Demandingness, Awfulizing, Frustration Intolerance, and Global Evaluation of Worth) in producing psychological disturbance. According to Ellis, Demandingness (consisting of demands and “musts”) is the core irrational belief which is at the heart of psychological disturbance. Awfulizing, Frustration Intolerance, and Global Evaluation of Worth are derivatives of Demandingness, each leading to psychological disturbance as well (DiGiuseppe et al., 2014, p. 37).

**Figure 1:**
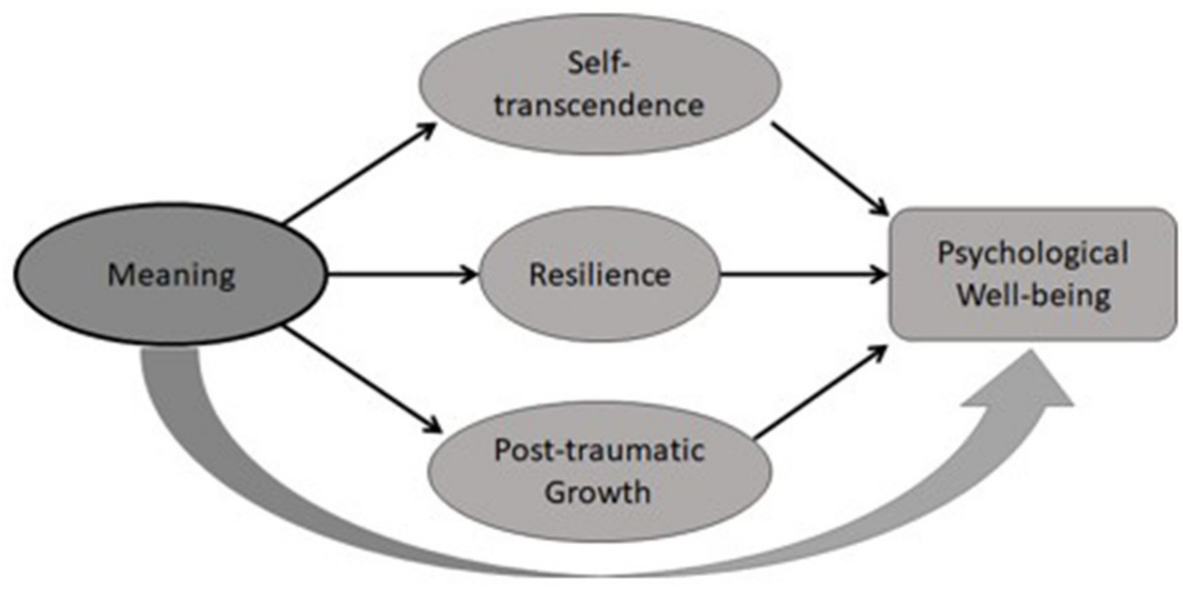
Logotherapy-based model of self-transcendence: meaning as core.

Logotherapy on the other hand, offers a coherent meaning-centered framework to enhance well-being, and to promote motivation for change and resilience, in order to face life's adversities in spite of suffering. When one has a reason, a purpose, or a “why” to live for, there is a higher probability to tolerate negative emotions such as pain, sadness, or disappointment, and in turn to develop perseverance and discipline, and to make necessary adjustments or even sacrifices for the sake of meaning. Along these lines, previous research suggests that meaning has been found to be associated with self-acceptance, emotional stability, resilience, and post-traumatic growth (e.g., Melton and Schulenberg, 2008; Breitbart and Masterson, 2016; Southwick et al., 2016; Weathers et al., 2016). We propose to complement the original Ellis's emotional disturbance model of irrational beliefs with Demandingness as the core irrational belief with a logotherapy based model of self-transcendence, with Meaning as the core beneficial belief, conceptualized in Figure 1.

Combining these two models may allow an integrative view of challenges and adversities experienced by many individuals during the current pandemic. On the one hand generating rational thinking by promoting flexibility, relativism, frustration tolerance, and unconditional acceptance (of self, others, and life) enables reduced distress and may facilitate the meaning discovery process. On the other hand, meaning discovery could act as a buffer against irrational beliefs by increasing well-being and facilitating self-acceptance.

Integrating rationality through REBT's emotional disturbance model and meaning through Logotherapy's model is likely to generate optimal beliefs, or “optimal sense-making.” We propose the term *optimal sense-making* as contextual. It depends on multiple individual factors such as the person's strengths, healthy resources and potentials, personally meaningful values, the specific situation at a given time, and all available opportunities and choices. Optimal sense-making may be defined as an intentional process of evaluating an adversity through both the lenses of rationality and meaning in order to motivate an individual in a given context to realize optimal decisions, choices and actions in accord to reason and his/her personally meaningful values, enabling him/her to tolerate the inevitable negative consequences, within a responsible, meaning-oriented and self-transcending frame. It is neither hedonistic, positive nor pleasure-oriented, but rather a realistic, flexible, mature, and meaningful way of thinking that is likely to help reduce distress, enhance resilience and generate growth by promoting acceptance, self-transcendence, and perseverance.

The importance of broadening the scope of existing models to support the healing and empowering of the clients is in alignment with Bruner's (1990) suggestions of moving beyond cognition and the importance of meaning-making and the cultural context. More recently, Beck et al. (2020) proposed the Recovery-Oriented Cognitive therapy (CT-R) to facilitate recovery and resilience in individuals with serious mental health issues. This is a strength-based model which emphasizes individuals' values, aspirations, and personally meaningful activities. At the clinical level, the integrative approach based on our optimal sense-making model presents various advantages such as to assist in moderating clients' resistance, increasing their tolerance to healthy negative emotions, and enhancing their motivation, openness, and perseverance toward long-term change.

To enhance motivation toward change, it would be useful to actively seek with resistant, less motivated or inconsistent clients, a powerful and meaningful reason which self-transcends them that may provide an 'anchor' to which they can strive and that will enable them to better tolerate negative emotions that may arise and maintain perseverance and hope. Along these lines, Ameli and Dattilio (2013) presented a clinical case to illustrate how self-transcendence (i.e., love for a spouse) can be used to motivate a resistant client suffering from generalized anxiety toward exposure and facilitation of cognitive restructuring. The construct of meaning based on values could be included as part of the REBT disputing process as well, by implementing logotherapeutic techniques such as dereflection (i.e., shifting the focus of attention from the problem toward meaning through self-transcendence) and attitude modification (challenging a negative attitude by activating the will to meaning through Socratic dialogue). That may enable to explicitly guide meaning discovery, and to assess the meaningfulness of the rational beliefs and the new effective philosophy generated by clients in order to direct them toward more optimal beliefs and philosophy (both rational and meaningful).

The concept of 'tragic optimism' (Frankl, 1986) could be implemented to help clients (1) turn suffering into human accomplishment, (2) turn guilt into a learning opportunity to change for the better, or (3) perceive life's finiteness as an incentive to use time wisely and to take responsible action (Frankl, 1959/1984). Along these lines, Lukas (1986) points out that in many occasions, behind a crisis, there is an opportunity to grow, and behind the suffering, a meaning. She recommends being cautious and tactful applying *logo-philosophy*, since it might be easier for the therapist than the client to discover the constructive or positive aspects available in face of the loss and suffering. These steps are particularly relevant for the current pandemic situation. To illustrate that, in the following section case illustrations and examples related to the pandemic are presented and discussed.

## Case Illustrations and Examples Related to the Pandemic

The concept of optimal sense-making based on the integrative model presented, could be valuable in assisting those affected directly or indirectly by the pandemic. Two examples are presented below to illustrate the integration of REBT and logotherapy toward optimal sense-making, at the clinical level. The first one is a real client therapy case. The second one is the real case of a pulmonologist who faced a near-death-experience during COVID. Finally, we include recommendations for working with dysfunctional beliefs held by distressed health professionals dealing with COVID 19 patients who have contacted us to receive therapy.

### Example 1: Clinical Case

A 67-year-old retired woman, a client with a history of depression, caught the flu during the lockdown period. She had a severe cough and felt extremely tired. Her doctor told her that she did not have the coronavirus and she was not in danger. She was unable to perform her daily activities and was put on bed rest. She was living alone. She had been a very hard-working client with a high level of discipline and perseverance.

As a first step, REBT was used to challenge the unhealthy and dysfunctional beliefs through disputation, in order to turn them into effective and helpful beliefs. Logotherapy was used as a second step to enhance a sense of meaning.

C: “This is terrible. Even though I do not have the coronavirus, we are in the middle of a pandemic, I am sick, alone, and I am not able to do anything. I don't have any strength to move on.”

T: I understand that this is a challenging situation for you. Considering the pandemic and the lockdown, it's not easy to be alone and sick in bed. However, I am confident that together, we can find ways to help you. What is the most important goal that you would like to achieve by the end of the session?

C: to feel better and get unstuck, to have some motivation to move on…

T: ok. Just to better understand your concern, what is for you the worst part, or the one thing that you would eliminate?

C: to be honest, being sick with the flu I guess. I am used to live alone and if I were not sick, it would not be that bad.

T: so for you being sick would be the terrible part of all?

C: correct. Being sick in this pandemic situation.

T: I understand. How do you feel, being sick this pandemic situation?

C: I feel like desperate…. I know the physician told me I do not have the coronavirus but this flu thing seems eternal to me. I am stuck!

T: I can imagine. How often do you feel desperate?

C: at least at some point every day. I feel like crying and not doing anything.

T: I understand how you could feel that way. To summarize, you believe that being sick is terrible in this pandemic situation. You feel desperate at least once a day and feel like crying and not doing anything. You are stuck, as if your flu was not going to go away. Does that seem right to you?

C: yes. I just don't want to feel this way anymore!

T: I totally understand. The good news is that we have learned together [in previous sessions] some concepts and techniques that could help you feel better and get unstuck. Going back to what we learned together about healthy and unhealthy negative emotions in face of adversity, despair like depression is an unhealthy emotion because it does not help you to get unstuck and move forward. What could be a healthy negative emotion that you would like to feel instead?

C: maybe sad, or disappointed?

T: good! Now let's see how to get there. Do you remember the ABC model we learned together some time ago and that you had the opportunity to practice with some of your issues?

C: yes. I was thinking about it. It's about your thoughts about the event determining how you feel, not the event. Therefore, if I feel an unhealthy emotion that means that I have to change my thoughts to feel better.

T: good summary! Our beliefs (B) regarding adversity (A) largely determine how we feel and how we act (C). As you well recall, when there is an unhealthy negative emotion at C like in your case here with despair, in order to change it, it's important to identify the unhealthy or dysfunctional belief behind it and turn it into a healthy belief that would lead to a healthy negative emotion. Do you remember how we did that?

C: yes, although I am not at my best today! I remember I had to question the belief to see if there were objective data that showed that it was realistic and if it was useful to reach my goal and see if there where shoulds or absolutism?

T: very good! You remember quite well. We use disputing to evaluate the belief based on three criteria: if it is realistic, based on objective data, logical meaning that it makes sense, and if it is functional meaning that it's useful or helpful to reach your goals. We also learned that shoulds based on absolutistic demands were unhealthy so we had to challenge them. Now, let's first examine your belief “being sick is terrible in this pandemic situation.” Before using the evaluation criteria, based on what we had learned previously, is there something that jumps at you?

C: the word terrible. I remember that we had to challenge it because it means as if everything is bad, like the end of the world!

T: you nailed it. Terrible would mean that everything is 100% bad for you right now or your whole life is ruined because you are sick in this pandemic situation. Now, using objective data, is that really true?

C: well, not really…. Not everything is bad or ruined. I just have the flu and I guess that I will get over it even if it's taking a long time. At least it's not dangerous like the coronavirus. In addition, my sons are wonderful, they are bringing me food and checking on me even in lockdown and I can do some little activities like listening to music or watching TV.

T: it is remarkable that you are able to see that not everything is negative and to point out objective aspects related to your issues that are not negative. This is evidence that your belief is not realistic. Your previous practice with disputing is paying off! Now, when we learned that terms such as terrible, horrible etc… are generally rooted in demanding absolutely somethings with shoulds, musts etc. Looking at your belief what are you demanding or imposing.

C: Yes, I remember that. I guess that I should not be sick in the pandemic.

T: right. Now, using the second logic-based criteria, is it logical to demand that?

C: No, it doesn't make any sense. I can't demand not to be sick, even if there is a pandemic.

T: right. This belief is not logical. You could wish or prefer not to be sick but it does not have to be that way even in a pandemic situation. Does that make sense to you?

C: yes. I remember that we talked about preferences and wishes. I have my index cards from other beliefs.

T: excellent! Now, let's focus on the third criteria, the functional one. How is this belief helping you to reach your goal of feeling better and being unstuck?

C: It's clearly not helping. It actually keeps me stuck and desperate!

T: right. This belief is clearly not helpful since it does not help you move toward your goals of feeling better and being unstuck. Now, summarizing, based on the three criteria, the belief “being sick is terrible in this pandemic situation” is unrealistic, illogical, and unhelpful. It's an unhealthy belief. As long as you hold on to this belief, you are likely to will feel desperate and stay stuck. Can you see that?

C: yes. If I want to feel better and get unstuck, I will have to change this.

T: good point. Now, let's see what an alternative healthy could be, one that would help you move toward your goals of feeling sad or disappointed instead of desperate and getting unstuck so you can move forward. Knowing that based on objective evidence, the word terrible is inaccurate, what would be a more accurate and objective term?

C: maybe unfortunate?

T: good! Now, what would be an alternative healthy belief?

C: It's unfortunate to be sick in this pandemic situation but it's not terrible because not everything is bad. The flu will go away and I will be able to get back to my activities.

T: excellent! You can also add if you want those positive aspects you mentioned about your sons and doing some activities. Things that you can do.

C: I like that idea. Seeing the positive is also helpful.

T: right. Now, let's check if that alternative belief fits with the three criteria: based on objective evidence, is it realistic, logical and helpful?

C: yes. It's all true.

T: how do you feel when you read that belief?

C: I feel like disappointed but at least I can see some light at the end of the tunnel. I am not that stuck anymore. However, to be honest with you, I don't feel motivated to do things even though I know I can do little things. It's like, what's the point… Am I being too difficult?

T: I totally understand. This does not have to do with being difficult and I am glad that you brought it up! Let's focus on the motivation issue. What do you think could motivate you?

C: I don't know… Something maybe that could at least matter to me…

T: I see. What is important for you in life, what really matters to you?

C: my family definitely. I have two wonderful sons and grandsons whom I love above all. The other one as you know is music, playing the piano, my creativity…

T: great! You also mentioned before that your sons are visiting you and helping you in spite of the lockdown situation. As a mother and a pianist, what could be a meaningful gift to your sons to show them your strength to do things and to move on in spite of being sick, alone, and confined?

C: good question! Maybe a song? But I am too tired to get on the piano…

T: how about just composing the notes in your head? I know you are a very talented musician and you told me that you had that ability!

C: yes, it's true. I can try to compose something in my head but what?

T: maybe something related to the pandemic. For example, a melody that represents for you the pandemic, which is an actual challenge right now. What are your thoughts?

C: I like that idea. It's like I can handle the pandemic situation, I can represent it! Composing the notes in my head will keep me busy even if it's not easy right now. Nevertheless, I enjoy doing this and knowing that it's a gift for my son is important for me! When I get better I will just play it on the piano and tape it.

T: fantastic! You see, when there is a meaningful task or project to fulfill, when we choose to do something for the sake of love, it's easier to find the strength and the motivation to do it in spite of adversity. This is what meaning is all about. Reaching beyond ourselves. This is a choice.

C: I agree. Composing that song as a gift for my sons shows my love and strength to them, even being sick in the pandemic. They would really appreciate it!

T: sure. It would be like turning this unfortunate or disappointing period into a creative project, out of love for your family! Can you see that?

C: yes! I feel more motivated now to do that, it's like a purpose.

T: Excellent! So, what are your most important takeaways from today's session?

C: that being sick in the pandemic is not terrible and that I can still find strength in music and love for my family to keep going!

T: very good! Now what could be your homework for this week?

C: I guess prepare an index card and tape it on my cell phone to listen to it several times every day or when I feel bad.

T: right. The goal would be to practice and implement the new healthy and meaningful belief we learned today and an index card would be a good reminder. Let's prepare together that card with all we did today.

**Final Index Card With the “Optimal Belief”:**

“It's unfortunate to be sick with the flu in this pandemic situation but it's not terrible. The flu will go away and I will go back to my daily activities. I can take advantage of this disappointing period to compose in my head the notes of a song that represents the pandemic for me. That would be a great gift of love to my family, the proof of my strength to move on in spite of being sick and alone in the pandemic. They would really appreciate it!”

**Result**: The client recovered from the flu and composed the song. She shared it with her family and some of her students and musician friends. They liked her song very much and congratulated her. She told the therapist “If I have been able to overcome this, I could overcome anything!”

**Analysis**: the above example shows how drawing on the client's unique talents to come up with a meaningful project, based on her creative and experiential values, and self-transcendence (i.e., a gift of love to her family) contributed to increase her motivation to move on and led to resilience. REBT was first used to De-catastrophize her thinking and come up with a helpful belief and a healthy negative emotion (disappointment vs. despair). Logotherapy was then used to turn disappointment into a motivating and meaningful project for the sake of her family. This integrative approach resulted in an optimal philosophy of life to enable her to better face her unique situation.

This case shows the added value of the integrative model based on optimal sense-making. Although REBT helped the client reduce distress and adopt a more flexible and accepting view of her situation, it was not enough to motivate her to move on. Logotherapy focused on client's strengths and values, helping her discover a worthwhile “why” that increased her motivation and well-being. It was easier to discover meaning at a second step since her thinking was already more flexible and healthier. The combination of both rationality and meaning led to acceptance, self-transcendence and resilience as proposed in our integrative model. This shows that optimal sense-making has the potential to be more efficient and powerful in long term than rational or meaningful thinking alone.

### Example 2: Real Case of a Pulmonologist Infected With Coronavirus Who Fought to Survive

Anooup Mahewareshi, a 59 year old pulmonologist residing and working in Southern California contracted the coronavirus in April 2020. He became seriously sick and didn't think that he would make it. He said goodbye to his family and friends. After spending 9 tough and challenging days at the hospital, he was finally able to survive Covid-19 and was released. The combination of several factors such as the treatment plan proposed by the doctors (including two long-term friends), openness to try a new medication, and a strong personal determination to fight and not give up were key. Anoop emphasized that “The emotional make-up matters. The support and prayers of friends and family matters. It is all a very humbling experience.” He also pointed out to the importance of the emotional support he received by his long-term Indian doctor friend of 35 years (member of his treatment team) through his words “you cannot give up on me. You have to fight” as a turning point for his decision to not give up. The Indian doctor who is a very committed and hardworking professional (working 12–14 h a day) returned to work his work at the hospital after only 2 weeks of recovery at home.

This case represents both rationality and meaning that seem to have contributed to survival. An analysis of an in-depth interview with Anoop is presented below to illustrate the integrative model.

**Analysis**: This case example shows how rationality, goal orientation with a plan and trust in significant and meaningful others could increase motivation for survival and hope in the face of death and hopelessness, leading to post-traumatic growth. Anoop described himself as already a strong, driven and logical person. However, he had decided to give up on life (“I kept telling myself that was like how a person in jails felt like. I was in an 8 by 10 room alone and with nothing to do…. I was feeling very hopeless and helpless. I told myself that it was it and I gave up. I wanted to let go and die”), and the emotional turning point occurred with the words of a loving, caring and trusted doctor friend “you can't give up on me.” He decided not to give up and disappoint a friend who knew him also very well. This has made him set a goal for himself, a worthy challenge. That meaningful friend helped shift his mindset to survival and hope. He was able to adopt an optimal form of thinking in his specific situation where friendship made a significant difference “I can't give up on my friend. This is not an option. I have the set goal of survival with a coherent plan and I will do whatever it takes to get there. Just say focused on your plan.” He actively acted against his initial catastrophic beliefs, and managed to cope in spite of all the challenges and pain involved. Not only was he able to face that tragedy without catastrophizing or adding horror to it, as Dryden (2020) highlighted but he also turned it into personal achievement. He grew as both a person and professional because of that traumatic near-death experience. At the professional level, he is now more compassionate toward his patients and is able to relate and connect better with them. He is also very generous with family visits to COVID patients and give a physician order to allow family members to visit despite the current restrictions. At the personal level, he testifies that he has gained more authenticity, more appreciation of life, and a higher awareness of life's finitude and the importance of time. He is more aware of the value of what matters in life and spends time doing what he likes and also feels closer to his family. Against all expectations, he decided to go back to the hospital after only 2 weeks of recovery. Although he is still at risk, when the thought of Coronavirus comes his mind, he just keeps going (“having gone through the emotions of near death experience… I would have never been able to imagine that without going through it. I learned that time is limited and now I do what makes me happy. I don't think as much as before. Now, I just do the things that I really want to do”).

This example is a good illustration of the integrative model: although rational thinking is helpful, it might not be sufficient to hang on to life, in a near-death situation. Anoop decided to survive only when a meaningful friend gave him a reason to do it, for his sake. Meaning, along with a coherent treatment plan helped him maintain rational thinking, avoid catastrophizing, and stay focused on his recovery plan and tolerate pain and discomfort. This shows how rationality based on Ellis's model and meaning as proposed in the logotherapy-based model interact to produce optimal sense-making which leads in this case to post-traumatic growth. Anoop seems to have interiorized that optimal philosophy since he was able to return to work by implementing a combination of rationality and meaning through dereflection in the high-risk hospital setting where he works: “when the fear of virus comes into my mind, I just keep going.” This shows how optimal sense-making could also lead to acceptance, self-transcendence and perseverance in long term as proposed by the integrative model.

### Working With First Line Health Professionals Struggling With the Pandemic

It is important to keep in mind that very few health professionals have previously confronted a global pandemic of this magnitude or have been trained to deal with one. During the first wave of the pandemic, in many countries, the citizens would applaud every night the health professionals and they were considered heroes. First line health professionals who attend many cases of COVID 19 patients and who seek therapy, often have the following types of beliefs: “I should save everybody. I can't make errors because it's terrible to fail. I will be a failure not a hero. I cannot stand this painful situation anymore. I am exhausted. I am not qualified to deal with this.”

There is a mixture of unhelpful thinking mostly related to self-downing and frustration intolerance, and a sense of hopelessness and despair. We suggest that it would be beneficial to integrate both REBT and Logotherapy strategies to provide them with psycheducation on both rational and meaningful philosophy to deal with the pandemic and its consequences. It would be important to emphasize authenticity, empathy, and tactfulness in the therapeutic process and adjust it to clients' individual characteristic and context, in order to be able to genuinely help and inspire them to an optimal form of philosophy to face the pandemic. An example could be the following: “I accept myself as a fallible and imperfect human being. Realistically, I will not be able to save every patient and that does not make me a failure, just human. My self-worth is not function of the number of patients I save. I am going to take care of myself all I can, in order to be able to give the best of myself to my patients. My vocation is stronger than this virus and tolerating this painful situation means not abandoning my patients, even if it's exhausting and tough. Choosing this attitude will help me to learn from my mistakes, gain more knowledge and build strength so I can move forward in spite of everything. My patients deserve it.”

An optimal philosophy in the face of the pandemic is not only realistic and helpful but also meaningful. In working with healthcare workers, unconditional self-acceptance through REBT in face of the global pandemic crisis is a very important goal as a first step. As a second step, Logotherapy could be implemented to highlight the meaning, purpose and values embedded in their work (e.g., asking why they have decided to choose their career, to emphasize their vocation and what it means to care for and serve their patients) and to identify a worthy goal to pursue. In this context, combining unconditional self-acceptance and self-transcendence may serve as key to perseverance, frustration tolerance, learning, and resilience. Highlighting the option to choose a heroic attitude, or the 'triumph of the human spirit' (Frankl, 1986) would be more helpful than focusing on the social concept of a hero. It is the choice of one's attitude that may also ultimately lead to post-traumatic growth and humility. It could also be beneficial to share with them and help them reflect on true stories of health professionals like the example of Anoop, who have survived the coronavirus and are back to the hospital dealing attending COVID19 patients. If dealing with awfulizing beliefs, it would be important to tactfully teach the difference between tragedy or crisis and horror as Dryden (2020) pointed out and the possibility to turn it into a personal accomplishment (learning, humility etc.) through logotherapy.

## Summary and Conclusions

In the current critical situation in which many individuals are finding themselves in new situations, often more limiting, a robust, profound, and efficient life philosophy is much needed in clinical practice to best support clients affected by the COVID19 pandemic. REBT and logotherapy are philosophically based, empirically supported, collaborative, and multicultural orientations with broad applications. They present multiple similarities that make them highly compatible and complementary. Building on previous insights proposed by Hutchinson and Chapman (2005) and Lewis (2009), a logotherapy-based model of self-transcendence with meaning as the core beneficial belief is proposed to complement Ellis' model of emotional disturbance with demandingness as the core irrational belief. Combining both models at the clinical level would lead to “optimal sense-making,” an integrative approach toward adversity which unites both rationality and meaning in order to assist clients to generate an optimal philosophy of life that would, in turn, empower them to face the pandemic with courage and without losing hope.

Recent studies have shown that higher levels of meaning in life correlate with lower states of anxiety and COVID-19 stress (Trzebiński et al., 2020), and that interventions aimed at finding meaning in life may help people to cope with the psychological effects of the pandemic (De Jong et al., 2020). The examples presented in this paper aimed to show the relevance of an integrative approach based on optimal sense-making at the clinical level. The first example is a real clinical case of catastrophic beliefs and despair that illustrates how integration through optimal sense-making could lead to motivation for meaningful action and resilience. The second example is a true case of a pulmonologist who was able to choose survival and perseverance by uniting a rational philosophy with meaning through friendship, which resulted in posttraumatic growth. Finally, suggestions for supporting health care professionals dealing with Covid-19 patients, based on the model, were presented.

A logotherapy-enhanced REBT based on optimal sense-making has the potential to increase the efficiency and effectiveness of the therapeutic process by integrating reason and meaning, self-transcendence and self-acceptance, flexibility and resilience. Furthermore, such approach includes the central concepts and wisdom of mindfulness-based therapies such as acceptance, love, gratitude, or compassion, without the challenges related to the Buddhist dogma or the Eastern philosophy and their consequent adaptation to the Western mentality. Once a rational attitude has been gained, the therapist can address the client's freedom of choice, values, strengths, and meaningful goals, as well as his or her sources of meaning in life (e.g., Lukas, 1998; Wong, 1998). Special attention should be given to the clients' own words, beliefs, and experiences, beyond symptoms of current situation, such as synchronicity and meaningful coincidence (Jung, 1969; Russo-Netzer and Icekson, 2020), transformative life experiences (TLE; Russo-Netzer and Davidov, 2020), and sacred moments (Lomax et al., 2011). Being mindful to such experiences may enable to elicit inner wisdom and to uncover “meaning cues” which in turn may broaden the client's perspective to see new possibilities and enable self-discovery (e.g., Lukas, 1998). Based on this foundation, the therapist can help the client to prioritize meaning in day to day living as a source of coping and thriving, in face of challenging times. In this sense, prioritizing meaning reflects individual differences in the extent to which meaning is implemented via the decisions individuals make about where to invest effort in the context of everyday life (Russo-Netzer, 2018a). Such prioritizing has been found to be connected with happiness, life satisfaction and gratitude among adults. This suggests that focusing on and prioritizing engagement in activities that are inherently value-congruent may serve as a tangible and concrete mechanism for instilling life with meaning and increasing well-being.

A major aspect of COVID 19 is not only the fear for one's health but also the social distancing it entails and the encompassing sense of uncertainty as to what will happen, how to cope and what could be the consequences. When we move beyond zero-sum structures and neat conceptual frameworks to a more holistic and integrative view, especially in face of the challenging and uncertain times of the pandemic, we are more capable of exploring the full and rich range of individuals' needs and experiences. Healing inevitably entails acknowledging and confronting the dark side of human existence, which reflects the dialectical coexistence of positives and negatives (Lomas and Ivtzan, 2015), and the principle of self-transcendence (Wilber, 1980, 2000; Wong, 2011). The brokenness, downfalls, and defeats, as well as the glorious highs and victories, are all important and valid part of our human wholeness (cf. Russo-Netzer, 2018b), and of our unique ability to leverage suffering into mental health and growth.

Finally, given that very few mental health care workers have been confronted with a pandemic of this magnitude, training therapists on this model during the current crisis could be challenging. A potential first step may be training practitioners expert in REBT and practitioners expert in Logotherapy on the proposed integrative model, and to set up pilot clinical trials with a target group of frontline care-givers (e.g., health professionals confronted with COVID patients) to assess the validity of the model based on its level of efficiency compared with REBT and Logotherapy alone. Based on these pilot trials, specific protocols could be developed to train practitioners and professionals to better handle future crisis. Encouraging evidence from recent multisite training and implementation of pilot programs during the pandemic support such directions (see, for example, Worley et al., 2020). Furthermore, given that video and virtual formats have been shown to be effective similarly to in-person care (e.g., Morland et al., 2017), this may be another flexible route for adapted therapists training during the current crisis.

## Author Contributions

All authors listed have made a substantial, direct and intellectual contribution to the work, and approved it for publication.

## Conflict of Interest

The authors declare that the research was conducted in the absence of any commercial or financial relationships that could be construed as a potential conflict of interest.
